# Energy System 4.0: Digitalization of the Energy Sector with Inclination towards Sustainability

**DOI:** 10.3390/s22176619

**Published:** 2022-09-01

**Authors:** Rajesh Singh, Shaik Vaseem Akram, Anita Gehlot, Dharam Buddhi, Neeraj Priyadarshi, Bhekisipho Twala

**Affiliations:** 1 Uttaranchal Institute of Technology, Uttaranchal University, Dehradun 248007, India; 2 Department of Project Management, Universidad InternacionalIberoamericana, Campeche C.P. 24560, Mexico; 3 Law College of Dehradun, Uttaranchal University, Dehradun 248007, India; 4 Department of Electrical Engineering, JIS College of Engineering, Kolkata 741235, India; 5 Digital Transformation Portfolio, Tshwane University of Technology, Staatsartillerie Rd., Pretoria West, Pretoria 0183, South Africa

**Keywords:** blockchain, energy trading, energy grid, smart grid, IoT, renewable energy, machine learning (ML)

## Abstract

The United Nations’ sustainable development goals have emphasized implementing sustainability to ensure environmental security for the future. Affordable energy, clean energy, and innovation in infrastructure are the relevant sustainable development goals that are applied to the energy sector. At present, digital technologies have a significant capability to realize the target of sustainability in energy. With this motivation, the study aims to discuss the significance of different digital technologies such as the Internet of Things (IoT), artificial intelligence (AI), edge computing, blockchain, and big data and their implementation in the different stages of energy such as generation, distribution, transmission, smart grid, and energy trading. The study also discusses the different architecture that has been implemented by previous studies for smart grid computing. Additionally, we addressed IoT-based microgrids, IoT services in electrical equipment, and blockchain-based energy trading. Finally, the article discusses the challenges and recommendations for the effective implementation of digital technologies in the energy sector for meeting sustainability. Big data for energy analytics, digital twins in smart grid modeling, virtual power plants with Metaverse, and green IoT are the major vital recommendations that are discussed in this study for future enhancement.

## 1. Introduction

**Figure 1:**
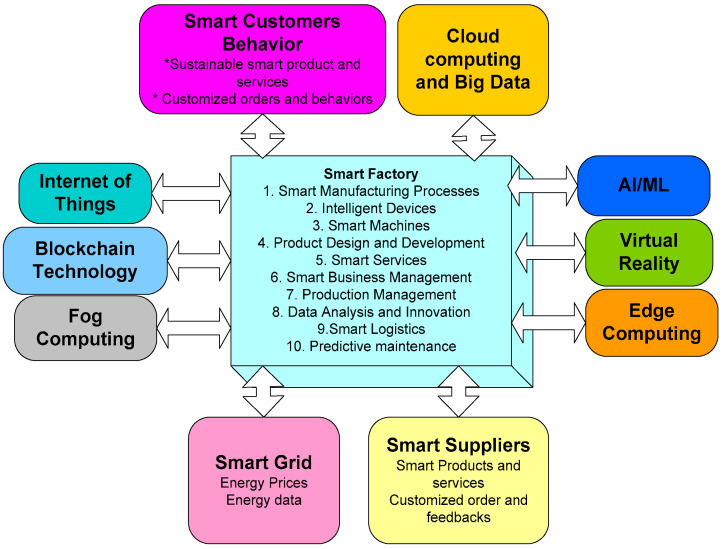
Architecture of smart factory in Industry 4.0 [5].

The United Nations’ (UN) 2030 agenda is to transform and provide a sustainable, affordable, and accessible environment for global citizens in an efficient manner [1]. Concerning Goal 9 of sustainable development goals (SDG), it is stated as “upgrading infrastructure and retrofit industries to make them sustainable, with increased resource-use efficiency and greater adoption of clean and environmentally sound technologies and industrial processes” [2]. Industry 4.0 is a digital transformation technology that plays a significant role in assisting organizations and society in moving towards building resilient infrastructure with sustainable industrialization [3]. A smart factory is one of Industry 4.0’s core paradigms, and is imagined as a future-state fully connected production system that functions primarily without human intervention by extracting, transferring, obtaining, and processing appropriate data to perform all needed tasks for producing all types of products [3]. The fundamental architecture of the smart factory in Industry 4.0 is shown in Figure 1, where the smart factory is amalgamated with distinct innovative technologies including the IoT, blockchain technology, cloud computing, big data, fog computing, edge computing, virtual reality, smart grid, AI, and machine learning (ML). The integration of these technologies in the field of the smart factory can accomplish applications such as smart manufacturing processes, smart machines, smart business management, production management, smart logistics, predictive maintenance, and intelligent devices [4]. The adoption of these technologies in the energy sector enables the realization of a smart system for the effective management of energy. Before addressing the significance of these technologies in the energy sector, we first present the problems in the field of the energy sector.

According to the International Energy Agency (IEA) 2019, smart factories utilize about 41.9% % of total global energy generated in 2019 and are followed by residential (26.6%), commercial, and public services (21.2%) [6]. These statistics conclude that the smart factories are demanding high levels of energy, which indeed increases CO_2_ emissions and presents challenges for achieving the clean energy and affordable energy goals formulated by the United Nations. In addition to this, SDG demand to minimize emissions by 55% by 2030, where IEA concluded that due to the absence of major policy action from governments, those emissions are set to remain around the same level for the next three years. However, as a result of global warming, the researchers suggest implementing green technologies such as zero-carbon green technologies (renewable energy), smart grids, and energy management [7]. To minimize emissions, renewable energy has been suggested and it has been adopted by the majority of countries. Along with this, the effective management of energy is necessary to minimize energy consumption and enhance energy efficiency. In the scenario of the energy system, there are four sub-systems, namely generation, transmission, distribution, and consumption, in which effective monitoring is highly required to properly identify the fault, manage peak loads, load forecasting, energy leakage, and quality management. Instability in any one of the phases directly impacts energy efficiency. However, the evolution of Industry 4.0 and its enabling technologies have proved to be capable in implementing a smart ecosystem in every area with digital monitoring; in the same manner, the implementation of these technologies in the energy system also boosts monitoring of the four sub-systems sustainably [8]. Transformation in the energy system also leads toward providing clean and sustainable energy, with innovative products and services for customer needs and consumption behavior [9]. Along with the four sub-systems, there are other areas such as energy trading, monitoring of electrical equipment, and energy storage systems that are significant areas in which Industry 4.0 can be adopted for different activities such as fault detection, fault diagnosis, lifetime expectancy of the energy storage system, and transparent trading of the energy [10].

Some existing surveys and reviews related to the adoption of Industry 4.0 in the energy system have been studied and compared. Reference [11] discussed the significance of digitization, digital twins, blockchain, and Industry 4.0 in the area of enterprises, where it concluded that energy companies need to implement blockchain and digital twins to meet social, environmental, and economic expectations. Reference [12] has addressed the machine learning application in the area of energy production, energy storage, and management. Reference [13] presented a detailed survey on the deployment of blockchain in smart grid scenarios to highlight the key security problems. Reference [14] emphasized the benefits of the IoT-enabled smart grid. Additionally, a detailed layered framework has been recommended in the study to characterize diverse IoT technology applications in the smart grid. Reference [15] carried out a survey in which they focused on the applications of blockchain with smart grids such as energy trading, intelligent energy management, security and privacy, and microgrid management; along with this, they have highlighted different frameworks and techniques.

The survey articles mentioned above have made outstanding contributions to the understanding of the state-of-the-art application of Industry 4.0 technologies such as AI, blockchain, and digital twins to smart grids. However, in these articles, only the individual technology’s impact on energy production, energy management, and the smart grid has been discussed. There are limited articles that integrate multiple technologies to discuss their impact on electricity generation, transmission, distribution, consumption, smart meters, electrical equipment, smart grid, microgrid, and energy trading. Based on this analysis, the study aims to examine the significance of IoT, AI, and blockchain in energy for different aspects, as IoT is the core technology for realizing Industry 4.0; along with this, AI enables prediction based on real-time data and blockchain for securing data and transactions. This article highlights the unique characteristics presented in the four sub-systems of energy smart grid, microgrid, and energy trading. This technology empowers the connection of different components of the energy sector on the digital network for better utilization and management of the environment [16]. The main contribution of the study is as follows:

1. The integration of IoT in four sub-systems of the smart grid, including generation, transmission, distribution, and utilization, is addressed. Architecture and the implementation of IoT-based smart grids with architecture as well as fog- and edge-assisted cloud architecture for the smart grid are presented with applications;
2. A discussion on the implementation of AI and blockchain to achieve demand-side management, forecasting of dispersed generation for the next day, forecasting of the smart grid network’s stability, and energy trading in enhancing the trading system in terms of security and management are discussed;
3. Big data for energy analytics, digital twins in smart grid modeling, virtual power plants with Metaverse, and green IoT are the major vital recommendations that are discussed in this study for future enhancement.

The organization of the article is as follows: Section 2 discusses the enabling technologies; Section 3 addresses the significance of IoT for energy in smart cities; Section 4 covers IoT integration in energy; Section 5 discusses the blockchain for energy trading; Section 6 discusses artificial intelligence in energy; and Section 7 presents discussion and recommendations.

### Methodology of the Study

**Figure 2:**
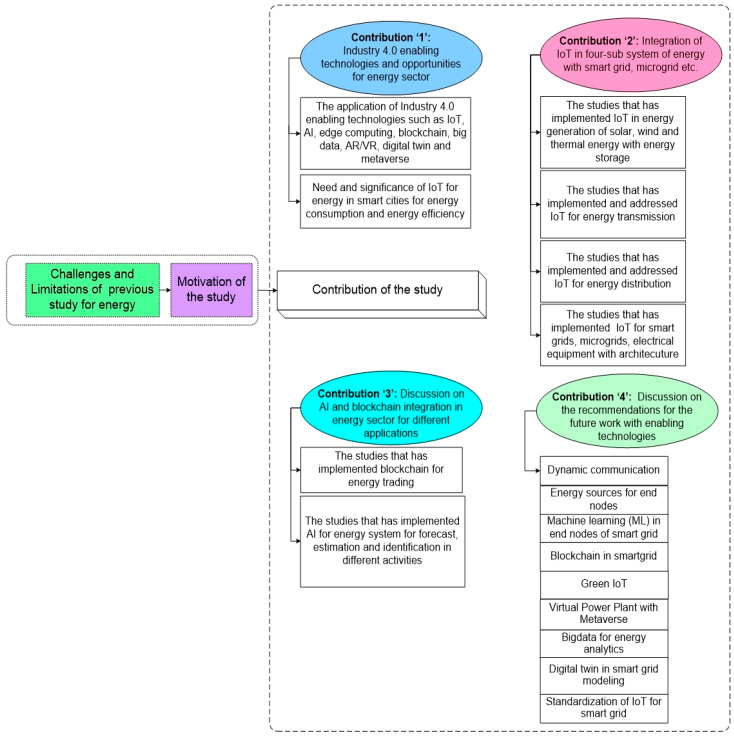
The approach of the study.

Figure 2 illustrates the strategy that is applied for the investigation of this study. Initially, the study examined the challenges of previous studies. Based upon these challenges, the relevant prior articles are analyzed to conclude that the challenges are properly addressed with appropriate discussion and identified as limitations. 

**Figure 3:**
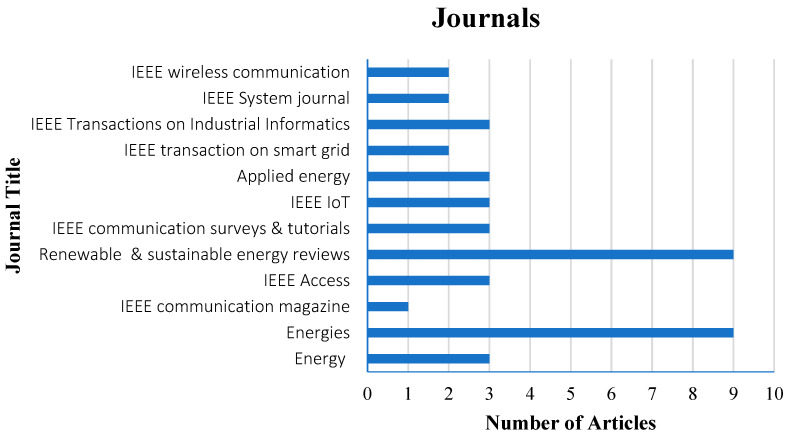
Articles distribution based on journal.

In searching for prior articles, the study has followed the above approaches, as this review focuses predominantly on the advancement of Industry 4.0 enabling technologies in the various activities of energy. The major research question is: “Which Industry 4.0 technologies are targeted in the energy system?” Based on this study question, we collected research articles from several databases such as Web of Science, IEEE Explore, and Scopus. To find an answer to the research question, we looked through papers having logical strings. The following strings are considered for obtaining publications: “Energy AND Industry 4.0”, “energy AND sustainability”, “energy management system” “Industry 4.0 AND digitalization”, “Energy sector AND digitalization”, “energy management AND IoT”, “IoT AND energy sector”, “Renewable energy AND energy management”, “Energy Efficiency AND Industry 4.0”, “Smart grid AND Industry 4.0”, “Energy trading AND Blockchain”, “Energy management AND Artificial Intelligence”, “Applications and Smart grid”, “Smart meter and domestic”, “Blockchain AND Energy”, “Information and Communication Technology AND Energy management”, and “Artificial Intelligence AND Energy efficiency “. The above search strings are used in the IEEE, Web of Science, and Scopus search filters. We searched articles on Web of Science and Scopus using the title, abstract, and keywords fields. In the instance of IEEE, we looked for articles that have abstracts. This study looked at papers from 2012 to 2022. Figure 3 illustrates the distribution of the articles in high-quality journals and the graph concludes that the highest number of articles are considered from the *Renewable and Sustainable Energy Reviews* and *Energies* (MDPI) in this study. These journals are followed by *Energy* (Elsevier), *IEEE Access*, *IEEE Communications Surveys and Tutorials*, *IEEE Transactions on Industrial Informatics*, *IEEE IoT*, and *Applied Energy.* The remaining articles in the journals are from conferences, books, and web links. Analysis of the obtained articles delivered the limitations of the previous studies and has motivated us to carry out the current study. Based on the motivation, the study examined more studies to frame the objectives in the form of contributions. Every contribution of the study is detailed and discussed with architecture and tabular representation.

## 2. Enabling Technologies

In current years, researchers have coined the novel term “Industry 4.0” [17]. The term refers to the fourth industrial revolution. The fourth revolution is referred to as internet use, also known as IIoT. The implementation of Industry 4.0 in the energy sector is necessary to integrate the enabling technologies for the realization of Energy System 4.0. Energy System 4.0 terminology denotes the age of digitalization in the energy field that signifies the distribution of digital infrastructure [18]. The significant components of IIoT are interconnectivity, data transparency, distributed decisions, and scientific assistance. In the following, the different enabling technologies are briefly discussed with their significance and applications.

(A). IoT:

The Internet of Things (IoT), often known as the Internet of Everything or the Industrial Internet, is a new technology paradigm envisioned as a global network of interconnected equipment and objects [19]. The basic architecture of IoT comprises three layers: the perception layer, the network layer, and the application layer. The sensors, actuators, and vision-based devices are the part of perception layer. The network layer inclues wireless communication protocols such as GPRS, 3G/4G/5G, Zigbee, Bluetooth 5, Bluetooth Low Energy (BLE), long range (LoRa), Sigfox, narrowband IoT (NB-IoT), and wireless fidelity (Wi-Fi) [20]. The application layer comprises web and mobile applications based on a cloud server for delivering vision-based services.

(B). AI:

In the digital age, businesses must respond quickly while maintaining high performance and competitive advantage. Over the last decade, a massive amount of data in various formats has been generated at a faster rate than ever before, accelerating technological advancement, and includes growing computing processing power and developing new techniques such as AI [21]. AI comprises any technique that allows robots to operate by emulating human behavior in order to obtain the best result or, in unclear settings, the best-expected result. Deep learning is a subfield of machine learning that deals with artificial neural networks, which are algorithms inspired by the structure and function of the brain. Deep learning algorithms are structured similarly to the nervous system, with one neuron connecting to the next and transmitting information. Deep learning models operate in layers, with a typical model having at least three layers. Each layer accepts information from the previous layer and passes it on to the next [22].

(C). Edge Computing:

Edge computing has been identified as a significant facilitator of IoT and mission-critical, vertical applications. ETSI defines edge computing as a novel technology that delivers an IT service environment and cloud computing capabilities at the mobile network’s edge, within a radio access network (RAN), and close to mobile subscribers [23]. Edge computing can be utilized for AI implementation at the edge network with different hardware such as graphics processing unit (GPU)-powered hardware, field-programmable gate array (FPGA)-powered hardware, and application-specific integrated circuit (ASIC)-powered hardware [24].

(D). Blockchain:

Blockchain is a shared and distributed database that holds a continually expanding log of transactions and their chronological order [25]. Transactions are accumulated into bigger formations called blocks, which are time-stamped and cryptographically connected to previous blocks, producing a chain of records that dictates the order in which events occur [26]. Blockchain 4.0 is primarily engaged with real-time applications such as public ledgers and distributed databases, where it incorporates Industry 4.0-based applications through smart contracts [27].

(E). Big Data:

Big data is defined as data for which the data amount, acquisition speed, or data format limits the ability to conduct successful analysis using traditional relational approaches, or data that can be effectively handled by employing important horizontal zoom technologies [28,29]. Big data enables firms to make decisions based on outside intelligence, as well as increase operational efficiency [30]. Big data analytics is a sort of advanced analytics that involves complicated applications powered by analytics systems that include aspects such as statistical algorithms and predictive models [31].

(F). AR/VR:

AR/VR is a technologically enhanced representation of the real physical ecosystem created through the application of sensory stimulation and digital visual elements. The top priorities of AR are to highlight certain physical world characteristics, increase knowledge of those features, and obtain sensible and approachable intelligence that can be employed in real applications [32]. VR is a computer-generated reality that immerses the viewer in their surroundings by using realistic-looking sights and objects. This environment is experienced through the use of a VR headset or helmet [33].

(G). Digital Twin:

A digital twin is a digital reproduction of a real product or component created by the combination of simulations and service metadata [34]. Data from numerous sources are included in the digital representation throughout the product life cycle. These data are constantly restructured and shown in a variety of ways in order to forecast current and future situations in both operational and design settings, therefore improving decision making [35].

(H). Metaverse:

Metaverse is a combination of the prefix meta (inferring transcendence) and the word universe, which depicts a hypothetical synthetic environment linked to the physical world [36]. The Metaverse is characterized as a virtual environment that incorporates physical and digital aspects, which has been made accessible by the combination of internet and web technologies, as well as extended reality (XR). 

## 3. Significance of IoT for Energy in Smart Cities

The IoT’s integration in smart cities is specifically aimed at controlling energy consumption; smart cities consist of smart public premises and smart home automation [37]. The lighting system in smart cities for transportation is embedded within the hybrid model and can be tracked and monitored with an IoT environment during the absence of a real-time system in the main electrical grid [38]. The lighting system in smart cities for the transportation network is established with a hybrid system, where the IoT ecosystem and the main electrical grid intervene for real-time tracking and monitoring. Smart home automation and smart building are the key elements of the smart grid. Trade and residential consumers have a significant proportion of the loads [39,40]. The performance of every individual is critical for the operation, as the overall consequences are not insignificant. The evolution of innovation in the devices encourages minimizing electrical consumption and enhancing efficiency along with sufficient energy in a building [41].

Although the incorporation of IoT into a building’s construction costs is higher, it provides significant advantages. Innovative solutions can be found through IoT use in buildings and homes that transform conventional structures and ensure a maximally efficient, convenient, affordable, and secure environment [42]. The study examined the energy-specific integration of IoT and realized that the key element in smart buildings is advanced metering infrastructure [43]. In order to transmit and receive real-time signals, all energy-supported devices and equipment are integrated with IoT technologies [44]. A wireless platform provides an opportunity to establish an access point to all the devices for sharing the information to the cloud data center. Moreover, some devices have access to long-term evolution (LTE) and 5G protocols so that the owner and the controller device have access to the mobile human–machine interface (HMIs) [45]. Based on the data from occupancy sensors and temperature sensors, the heating, ventilation, and air-conditioning system (HVAC) can be successfully adjusted, and it leads to a significant reduction in energy waste [46]. Vehicle-to-grid technologies (V2Gs) can also be programmed and operated with the assistance of IoT gateways for absorbing or powering from the grid [47]. The storage unit will supply the grid at its peak, depending on the building’s load pattern and capacity. V2Gs can be charged for real-time electricity price tracking automatically at midnight and are able to sell excess stored energy at peaks to the main grid [48]. Thus, a smart cloud computing environment can be materialized with IoT technologies and information and communication technology (ICT) with high bandwidth and high-speed communications infrastructure.

The use of a CHP (combined heat and power) system can significantly enhance the efficacy of a building’s energy management [49]. Regarding an energy hub that takes into account the electricity and gas prices given by the advanced metering infrastructure (AMIs), the optimal time to generate the CHP unit must be determined by the energy management system in the building [50]. The concept of an energy system considers gas and electricity costs recorded by AMIs and the optimal times for generating the CHP are provided by the energy management scheme of smart buildings. Furthermore, the Zigbee protocol is embedded in AMI for automatic control of modern devices and appliances. Concerning electricity costs, for instance, a dishwasher may automatically begin at midnight [17]. The integration of IoT in AMI transforms the metering infrastructure into a real-time system for obtaining the data of load on a distinct time scale [51]. In addition to automatically responding to demanded household appliances or devices, the end-users may spontaneously perform certain DRPs. Therefore, the user can control various devices and devices employing computer-specific interfaces or AMIs such as smartphones and tablets more conveniently by using an IoT-based communications infrastructure [51]. Table 1 clearly illustrates a few more applications related to IoT integration in the energy sector.

A large proportion of electricity demand is attributed to industrial consumption and industries are classified as sensitive loads that need more stable electricity supplies than domestic, business, and agricultural consumption [52]. Moreover, in terms of tensile regulation, frequency stability, and harmonics, industries need better quality power. Different types of charge are used in this sector, on the other hand, such as power electronic devices, induced machinery, and synchronous engines [53]. Control of power in this section is extremely important in this respect and, in addition, the owner’s special attention must be paid to the interaction of industrial loads with wholesale or retail power markets. Table 2 illustrates the technical specifications of wireless communication technologies that are widely implemented in IIoT.

## 4. IoT integration in Energy

As discussed in Section 2, we consider the integration of Industry 4.0 enabling technologies in energy. In this section, we have discussed IoT in the generation, distribution, and management of smart grids and microgrids.

### 4.1. Electricity Generation

Resource generation management was previously monitored utilizing local control devices. System operators have a low remote control capability, and several actions have to be completed with commands or instructions from a local operator [55]. Moreover, for many reasons, the asset management of power system generation becomes more advanced [56]. The first reason is renewable energy resources penetrate energy systems as a great source of inconsistency [57]. Second, electric vehicles will be widely utilized and will affect planning for electricity generation [58]. Third, loads are increasingly involved in the response to demand resources, which is very much in line with unsafe hourly prices of electricity [59]. The cost of electricity is associated with various items including the power market and instant fuel price. Moreover, small-scale distribution generation (DG) will gain extensive dominance in the future as a virtual power plant [60]. In addition to existing grid limitations, the operator must address a high level of uncertainty and volatility in microgrids that may, in some cases, impose load shedding or restriction.

**Figure 4:**
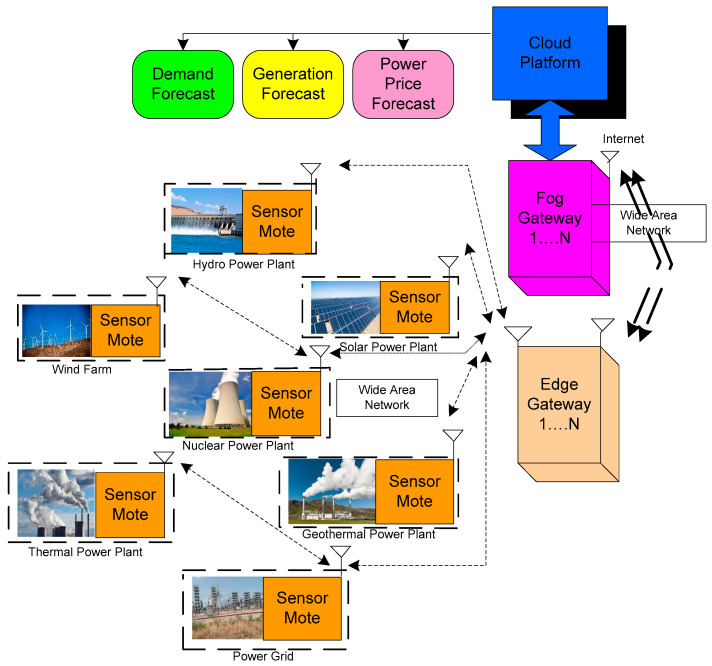
IoT based on Real-time monitoring of generation stage using edge and fog gateway.

IoT technology enables the resolution of issues by avoiding the procedures and preserving the safety, steadiness, consistency, and environmental affordability of the power system [61]. Variations and production in demand and supply can be tracked remotely and precisely in an IoT-based smart grid, and the operator can have more detailed monitoring of the grid [62]. IoT generation-level technologies are designed to integrate a multitude of sources of energy such as hydro, coal, gas, oil, nuclear, wind energy, solar energy, geothermal, and marine power to strengthen production performance and preserve the powerful and static security of the electricity system [63]. Moreover, energy storage systems are employed to restore disparities caused by a range of sources of uncertainty that IoT infrastructures can affect. The realization of IoT in the generation stage of the smart grid is illustrated in Figure 4. The architecture presents the significance of integration of fog and edge computing with the cloud for the smart grid. ‘n’ number of sensor motes will be deployed in the geothermal power plant, thermal power plant, solar power plant, hydropower plant, and wind farm for sensing environmental parameters.

The sensor mote is the combination of distinct sensors and a wireless communication module. The wireless communication module communicates the sensory information of every plant to the edge gateway. The edge gateway is the integration of edge computing where it executes the algorithm on the received sensory data and sends necessary instructions to the sensory mote. The edge gateway assists to overcome the fault or errors quickly. The edge gateway also predicts the demand, generation, and power price. The connectivity between the edge and fog gateway is established with the assistance of a wide area network (WAN). Fog computing is an amalgamation of two significant components of data processing, namely cloud and edge. The concept of integrating cloud computing is associated with edge computing for more reliable and faster data processing. The wide implementation of the smart grid triggers fog computing to act as an optimal tool for data processing among grid operators, energy providers, and consumers. Thereafter, the connectivity between the fog gateway and cloud platform is initiated through the internet.

**a**. **Wind energy:**

Wind energy is rapidly evolving in terms of productivity, and energy professionals have set goals for wind energy deployment in the future [64] The major impediment to the growth of wind energy is natural resources. Wind units have high precision for supporting demand, and extreme disparities may endanger system security [65]. Real-time operation enables the establishment of the remaining electricity system for repaying the volatility without having to endure strong ramp rates. Moreover, better working relationships with energy storage systems can only be preserved if the wind units and the energy storage unit exchange real-time data [66]. IoT technologies and ICT facilities allow operators of wind farms to conduct precise maintenance schedules that prevent enormous detrimental effects, and such a timetable can be carried out by machine learning and data mining [67,68]. In exploiting wind power, IoT’s need is to immediately collect and analyze information related to wind turbines and wind farms. 

Currently, two problems must be overcome: Offshore wind farms have obstacles in the form of data transmission delay and bandwidth limitations to the transmission of information for remote sites [69,70]. The implementation of critical data obtained and analyzed in real time can be made quicker or automated to make a decision (such as to shut down the turbine to prevent cascade). The deployment of wind energy IoT systems demonstrates the need for broader economic, safe strategies in the design, operation, and installation of wind farms and the conserving of turbines [71]. A wind turbine consists of major components, namely the yaw system, tower, foundation structure, rotor hub, blades, drive shaft, brake system, doubly fed induction generator (DFIG), wind sensor, transformer, nacelle, and the central controller [72]. 

There are several sensors and actuators in the controller layer. The sensors can inform each essential component’s health and performance. The control system manages components with a series of actuators and handles them [72]. The sensors are divided into five layers: electrical, environmental, mechanical, temperature, and fluid sensors. The controller obtains the sensors’ data and transmits the hydraulic electric and mechanical commands and instructions employing power amplifiers [73,74]. The instructions are conducted with a transmission controller system, linkage controller system, motors, switches, pitch angle control, fans, pistons of positioning, and heaters. The cyber–physical devices integrate into the network infrastructure to interconnect the physical layer of wind turbines and the cyber layer, encompassing the network, tracking, and SCADA systems [75]. Network refers to a reliable connection between controllers and sub-systems for the transmission of data and controller signals as well as the connection of smart and deeply integrated equipment in a wind farm. 

The network plays a major role in allowing data transmission and control signal between the monitoring center, actuators, sensors, controller, and data storage stations in real time. The design of the network depends greatly on local conditions, particularly for offshore wind farms. A remote terminal unit (RTU) is configured to link each turbine to a local area network. The condition monitoring system is established by the local area network of wind turbines integrating with a SCADA system, which allows for the pre-definition of faults in wind turbines and the prevention of chain reactions in a wind farm [76]. Condition monitoring systems hold the stability through under voltage ride-through, fault ride-through, and low voltage ride-through schemes.

For implementing real-time monitoring, wind turbines are embedded with IoT, machine-to-machine (M2M) communication, wireless sensor networks, and cloud servers [77]. Computer-aided interfaces or mobile human–machine interfaces (HMIs) are integrated for monitoring the wind turbines in real time. The information technology-based control system is more expensive than traditional SCADA systems currently; however, it is more diagnostic because of the higher frequency of information and the maximum sampling rate. The IEC61400-25 standard is developed to ensure the extendibility and autonomy of the data exchange gateway, diagnostics, and standardization [78].

**b**. **Solar energy:**

Global warming and environmental issues directly impact the establishment of world energy demand for renewable energy resources, and power generation from renewable energy has the highest potential [79,80]. This source is also considered to play a leading role in future clean energy systems. Solar energy is also known as radiant heat and sunlight and can be harnessed using photovoltaic technologies that are ever developing. Photovoltaic technologies can be dispersedly deployed as decentralized or concentrated solar systems (CSP) [81]. A solar panel, cabling, switches, assembly system, and inventors are essentially photovoltaic systems, and the battery storage unit can be used with those accessories.

Advanced photovoltaic systems have more advanced technologies for efficiently extracting solar energy, including an anemometer, maximum power point tracker (MPPT), and additional task-specific accessories [82]. Unlike conventional photovoltaic systems, concentrated photovoltaic technology comes with curved mirrors and optical lenses that focus on irradiation for achieving remarkable efficiency in the solar cell [83]. In order to increase efficiency, a cooling system is typically incorporated into concentrated photovoltaic systems. Concentrated photovoltaic and CSP systems are suitable for maximum irradiation zones (namely the Golden Banana region in Europe and Sunbelt region in the U.S.). The traditional photovoltaic systems are also utilized to produce end-user-distributed systems including building-integrated or rooftop solar production, as the cost of the capital per kW is considerably less [84]. At present, grid-connected rather than standalone approaches are significant contributions of PV systems. The power output of PV systems depends primarily on room temperature and the intensity of sunlight radiation. Dust and shading trigger a dramatic decrease in the output power, and it simultaneously worsens the photovoltaic system’s performance [85]. Moreover, the efficacy of the photovoltaic system declines at maximum temperature.

When it is partially clouded, the MPPT inclines the panel directly toward the brightest part of the solar radiation of the sky [86]. The existence of a storage plant is crucial because solar power has to store the energy for distribution during requirements. IoT refers to the synchronization of analogs with solitary identification equipment, mechanical machines, objects, and computers [85]. IoT allows communication of the information along the network by eradicating the rift between information technology systems without human intervention [87]. IoT assists in communicating all data from photovoltaics in real-time, remote supervision during preventive maintenance, and fault detection of the photovoltaic system [88]. Furthermore, grid-scale coordination requires real-time, IoT-compatible communication for supervising the uncertainties in solar power generation and storage plants.

Since photovoltaic systems are far away from the city and few are located in the desert, in order of prevent losses and failures, it is challenging for humans to monitor all photovoltaic panels because frequent visits to the photovoltaic plant and the recording of the operative data are needed. These human failures take a long time to address and are not easily identifiable at certain times. So, the photovoltaic panels must be equipped with a continuous real-time monitoring system for monitoring the parameters of the photovoltaic system and logging the necessary data on a cloud server [89]. The logged data can be utilized for enhancing the performance of the photovoltaic system and identifying the causes of poor performance

**c**. **Thermal generation:**

Thermal power plants are currently an essential component of all energy systems. These kinds of units guarantee grid operation reliability and resilience. Indeed, due to environmental issues, thermal generation is being sought in future power systems to replace traditional heat plants with renewable resources. They also function with minimum efficiency and low flexibility. Currently, gas-fired units are considered costly generators. These issues demonstrate that, in this sector, IoT is probably the least deployed in comparison to other components of electricity grids, namely distribution, demand, and generation. In two senses of the word, however, IoT can play a key role in realizing the output status of the generators, transformers, and tap changers together with the power injected into every individual branch of the system’s central control center. The IoT infrastructure can, thus, easily access such data in real time. In addition, traditional steam power stations have a range of components and elements. In order to perform overhauls and preventive maintenance events for mitigating the risk of unplanned failures, the health status of all engines is recorded and monitored automatically.

**d**. **IoT for energy storage facilities:**

Electricity markets are gradually moving towards an intelligent environment that allows for smart and autonomous generation to cooperate with many providers and customers. The reason is that, in the last two decades, renewables have been penetrating an incremental trend that increases the level of grid operation uncertainty. Energy storage technologies contribute to the dispatch ability by resolving the imbalances of uncertain renewable resources. A grid-scale battery energy storage system (BESS) consists of a control system, a battery bank, protective circuitry, power electronics interface for AC–DC power conversion, and a transformer to alter the BESS output to the transmission or distribution system voltage level [90]. Traditional uninterruptible power supplies are installed in series with their loads, whereas a BESS is often connected to the grid in parallel with the source or loads it is supporting. A power conversion unit is typically a bi-directional unit capable of four-quadrant operation, meaning that both real and reactive power can be delivered or absorbed independently according to the needs of the power system, up to the rated apparent power of the converter.

The incorporation of IoT and the massive data package require high complexity while boosting performance at autonomy levels. A sensible balance between complexity and performance must always be established. Energy storage devices are classified into several types including small-scale frequency regulation, bulk energy time shifting, frequency stability, and power reliability [91,92]. So far, diverse energy storage technologies are established for numerous applications. Compressed air energy storage and pumped hydro energy storage are the two significant technologies applied for bulk energy time-shifting [93,94]. Moreover, a few emerging energy storages are presented, such as liquid air energy storage, advanced rail energy storage, underwater compressed air energy storage, ocean renewable energy storage, and the blue battery in green power islands, which are tested on a pilot scale in large-scale applications [95,96,97,98,99].

In small-scale applications, such as frequency and power quality regulations, distinct types of batteries, flywheels, and fuel cells are implemented. Energy storage plays a key contribution in enhancing the flexibility and reliability of power systems. Their uncertain and intermittent nature is a major obstacle to the greater use of energy resources. By using power storage facilities, the challenges of such qualms can be mitigated. In order to avoid undesirable cuts for excess-generating potential or detrimental deficiencies, a real-time interconnection between these units is essential. In order to facilitate the joint operation of solar farms or parks with energy storage grids, IoT infrastructure can upgrade this condition, enabling all types of units to be more profitable. The effect of IoT on the better use of small-scale energy storage systems such as batteries is utilized in frequency stabilization microgrids.

### 4.2. Electricity Transmission

**Figure 5:**
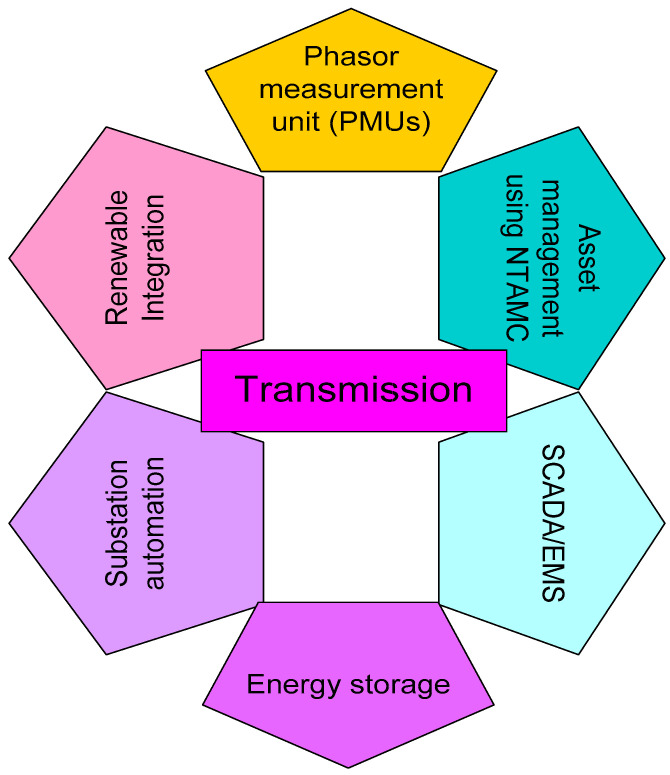
Application of IoT for electricity transmission.

The transmission level acts as the bridge between the production level and distribution level. Transmission has a prominent role in electricity systems that ensures reliable demand supply [100,101]. Firstly, we discuss IoT’s role in improving congestion management and, secondly, we discuss IoT’s effect on system safety conservation [102]. The installation of IoT and smart electronic devices into the transmission sector encourages the operator to monitor the electrical status of transmission lines, including perturbations [103,104]. Figure 5 illustrates the application of IoT in electricity transmission and they are named as substation automation, energy storage, SCADA/EMS, phasor measurement units (PMUs), assets management using NTAMC, and renewable integration PMU, which determines voltage and current angle and magnitude on a particular line point by employing the GPS for synchronization of time. A commercial PMU version will report measures of approximately 30–60 measurements per second with high temporal resolution and enables engineers of the power systems to examine the dynamic events in the power system [105]. With conventional SCADA, such a rapid and precise measurement is not possible every 2 or 4 s. The evolution of non-GPS reference-time micro-synchronous PMUs executes 120 samples per second for combating cataclysmic blackouts. PMUs transmit the data to demonstrate high-precision reactive and active power pathing over the transmission line, thereby improving system visibility. The real-time monitoring of power flow over the transmission wires assists the operator in automatically accomplishing traffic in congested power systems or areas, especially in an emergency [106].

Hard winds and strong snowy conditions lead to lines that cause the asymmetrical pulling force to be exerted on lines, which may lead to towers leaning [107]. These factors harm overhead lines that increase operational risk. Maintenance and remote monitoring are the key challenges that arise in the large transmission grid. Integration of IoT will alleviate the damage caused by natural phenomena of this kind. Data from advanced sensors installed on the tower conductor of the line must be obtained accordingly. The data are transmitted to the synchronous node device through optical fiber or wireless communication to the central control center. Tower deviation sensors, sync node devices, meteorological sensors, wind speed sensors, conductivity sensors, and current leakage sensors are IoT devices that can be embedded into the transmission grid [108,109]. These devices can help to improve real-time driver tracking, insulation, and towers.

### 4.3. Electricity Distribution

**Figure 6:**
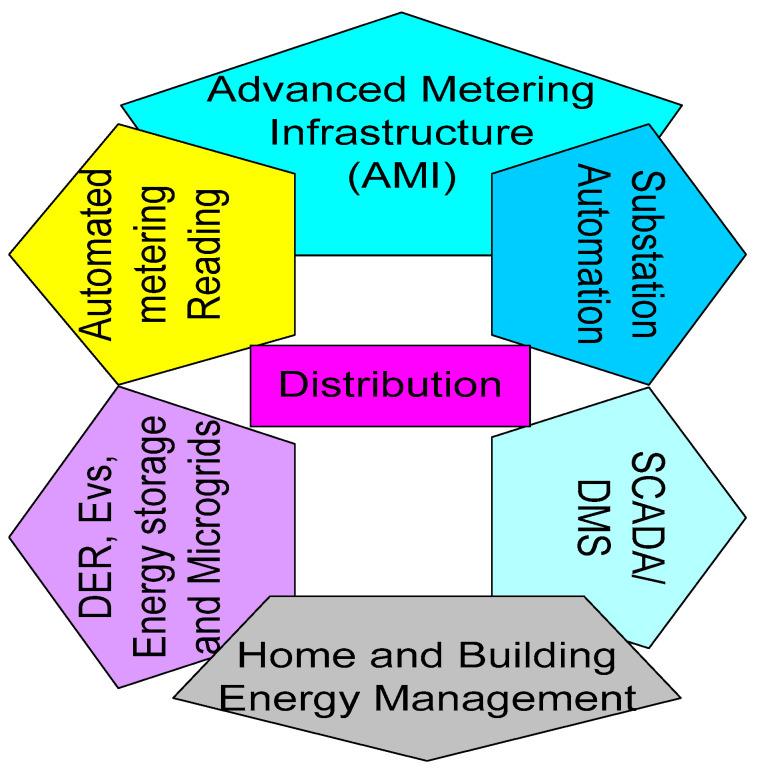
Application of IoT for electricity distribution.

An intelligent distribution grid must be fitted with IoT infrastructures that monitor critical components of the distribution network. This is shown in Figure 6. Installing AMIs is the first phase in implementing an intelligent consumer distribution grid and the most critical component of AMI systems is the communication network [110]. It provides a two-way, coherent, and safe connection among servers, data collectors, counters, customers, and recipients. Online supervision of consumption, fault detection in low-voltage transmission lines, smart control of energy consumption and generation, self-healing schemes, power loss management, emergency demand response mechanism, and remote monitoring during natural disasters are applications of integrating IoT in electricity distribution [111,112].

Furthermore, the data collected from every feeder need to be digitized by local ICT networks so that the distribution authority can monitor the distribution grid [113]. A self-healing scheme is a prominent and essential system for the grid in the future for enhancing the reliability of the grid. Self-healing schemes need to be implemented in real time for retrieving the appropriate and desired functionality. Renewable energy sources need to be more efficiently operated in the distribution networks by establishing a distributing network for monitoring supply and demand. In this context, the operator is required to be instantly generated with the assistance of IoT-based AMIs, and appropriate controls based on predetermined settings have to be carried out automatically [114]. An IoT network deployed for smart meters must be leveraged for other minima and throughput, fewer power applications including home/building automation and distribution automation, street light automation, demand response, etc.

### 4.4. Smart Grid

A smart grid is the amalgamation of the four sub-systems, namely power generation, power transmission, power distribution, and power consumption [115]. The application of IoT enhances the four sub-systems of the smart grid. In the field of power generation, IoT is employed for monitoring and controlling solar power plants, geothermal plants, and wind power plants, as well as evaluating energy consumption, equipment health, gas emission and pollution discharge, power correction, power reading, and power prediction. IoT provides an opportunity in power transmission for health monitoring of transmission lines, detection of tilted transmission towers, as well as monitoring of power protection circuitry and safety against vegetation through swarm drones and smart patrol. Monitoring the level of oil in transformers, power theft, the health of transformers, operation and equipment management, and RYB phase detectors of LT pillars all use IoT in the field of power distribution. Advanced meter infrastructure, home automation, charging infrastructure, information management of electric vehicles (EVs), power demand infrastructure, energy efficiency monitoring, energy consumption data collection and billing, load balancing and controlling, multi-network consumption, integration of distributed energy resources, energy profiles for home, and hardware chatbots for energy profiles of users are possible with IoT in the field of power consumption. Revolutionizing the energy grid into a resilient infrastructure with groundbreaking technologies is represented as a smart grid. A smart grid is realized with bi-directional wireless communication protocols, computer processing, and a control system. The advanced technologies that incorporate smart sensors include phasor measurement units (PMUs) that assist operators in assessing grid stability, smart digital meters that give consumers smarter information and remotely record disruptions, electrical relays that perceive and recover from substation faults automatically and re-route power around problems, and batteries that shunt power around problems [116].

**Figure 7:**
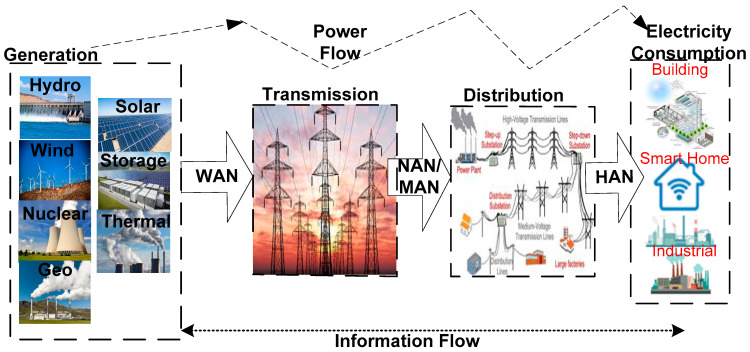
Architecture presenting power system, information flow, and power flow [117].

Figure 7 illustrates the smart grid architecture whose details present the power system, power flow, and information flow. Here, the four sub-systems of the smart grid are integrated with three appropriate distinct kinds of communication networks, namely wide area network (WAN), home area network (HAN), and neighborhood area network (NAN), for power generation, power transmission, power distribution, and power consumption [117]. WAN act as the cornerstone for enabling communication between gateways or aggregation points. WAN is the network integrating energy generation sources such as geo, thermal, wind, solar, nuclear, and hydro to monitor and control the generation and transmission systems remotely from any location [118]. WAN is the combination of two interconnected networks including a core network and a backhaul network. Core networks enable the delivery of facilities of maximum data rate and minimum latency communication to utility control centers via cellular communication and optical fiber communication. The backhaul networks offer wired and hybrid fiber–wireless networks with broadband links and monitoring devices to NANs. Further installation of video camera surveillance systems in smart grid management ensures the security of assets, fire alarm, and stable activity.

NAN is the second layer of the smart grid, and it comprises digital meters relating to various HANs and further communicates to the data collector devices that interconnect NANs to WAN [119]. This layer initiates communication for connecting distribution substations and field electrical appliances for power distribution systems. The gateway present in the NAN enables the system to obtain the energy consumption data of smart meters in HAN and further transmits it to the utility companies over WANs [37]. Generally, the network topology of NAN consists of two distinct gateways, namely the HAN gateway and the NAN gateway. The NAN gateway is capable of connecting to numerous gateways, where it acts as a wireless access point for providing single-hop communication to HAN gateways. HAN gateways are the medium for communicating the consumption data of numerous smart meters through wired and wireless communication technologies. However, several parameters, namely maximum transmission range and interference-free and low-power consumption, are essential for a communication protocol that integrates reliable connectivity [120].

HAN is a significant layer in the architecture that transmits and demands the power requirements of home appliances and smart appliances, including renewable energy sources and electrical vehicles [121,122]. HAN is installed in smart meters of homes, industries, and commercial buildings, either in mesh topology or star topology. Here, the gateway is interconnected with smart meters for collecting the power consumption data and transmitting with the most significant and appropriate wireless technologies, including Zigbee, powerline communications, and Wi-Fi. HAN performs bi-directional communication regarding demand response management where forward communication transmits the real-time information of smart meters and energy consumption to the NAN [123]. The backward communication is represented as a central node and can obtain the electricity pricing information through the NAN; communication also performs trigging events in the necessary situation in home appliances [124].

(*a*) *Hybrid fog-assisted cloud architecture for smart grid*

The combination of diverse IoT devices in the four sub-systems of the smart grid generates a tremendous intensity of heterogeneous data [125,126] and it is challenging for networks to tackle huge amounts of data when it hits the cloud server due to limitations such as traffic handling of low-latency data and limited resource distribution [127,128]. To overcome these limitations, fog computing can be employed as it integrates application-specific logic in intermediate infrastructure along with edge devices and remote clouds [129,130].

**Figure 8:**
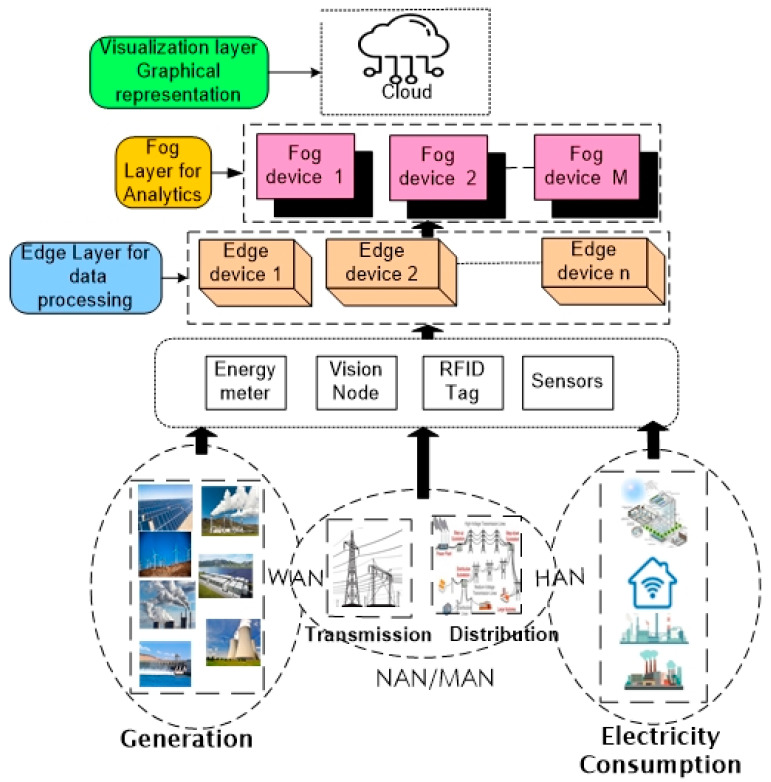
Hybrid fog-assisted cloud architecture for smart grid [131].

An architecture to monitor and control the computational task of the smart grid is proposed by [131] and is shown in Figure 8. The architecture is capable of performing analytics on smart grid data in fog and edge layers. In the first layer, the basic infrastructure of the smart grid sub-systems generation, transmission, distribution, and consumption are embedded with IoT-enabled sensor nodes to sense the physical parameters. In the second layer, the physical devices that are deployed in households and industries are energy meters, vision nodes, IoT-based sensors, and RFID tags. As fog motes and edge motes are connected to devices, it is feasible to perform analysis of real-time data for checking latency, quality of service, and reliability. The cloud layer is the final layer and a major layer of the architecture, as it provides a platform to visualize and monitor the real-time events and data of the smart grid from any location through internet connectivity.

(*b*) *Applications of IoT-based smart grid*

**Figure 9:**
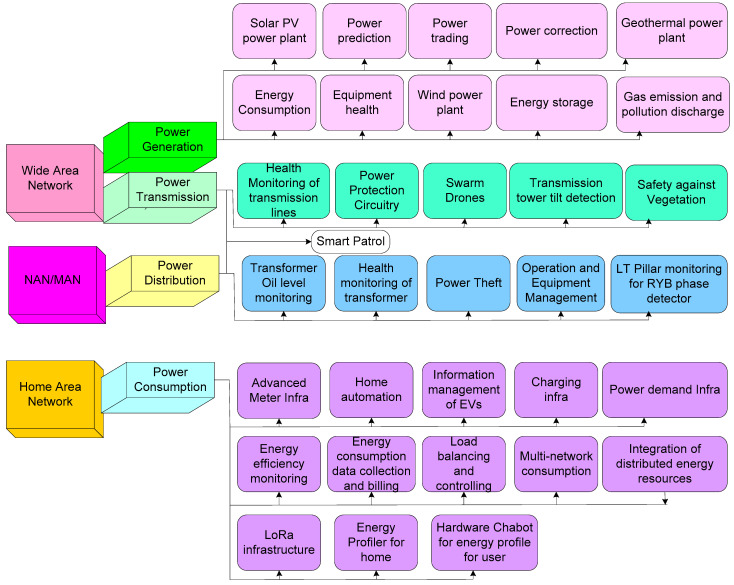
Potential application of IoT-enabled smart grid.

There are many existing applications of IoT-based smart grids (represented in Figure 9) and previous applications, with the addition of a few more applications of IoT-based smart grids, are addressed in this section. The applications are categorized concerning the four sub-systems and three communication networks (WAN, NAN, and HAN). In power generation, the possible applications concern monitoring energy resources, namely solar power, wind power, and geothermal power plants through WAN [132]. Additional applications include monitoring energy storage units, power prediction, power correction, and power trading. The monitoring of the health status of the equipment is achieved with the assistance of IoT. In the field of power transmission, transmission line plays an integral role in possible applications in the area of rigorously monitoring the tilt position of transmission power and health status monitoring of transmission lines. Generally, in a few specific cases, it is challenging for authorities to patrol the conditions of the transmission tower. Integration of IoT enhances the protection of transmission towers from natural disasters, unsafe construction, and the growing of vegetation around the foundation of transmission towers. Here, IoT smart patrol and swarm drones have the potential to reach the destination of the transmission tower and sense the anomalies of the transmission tower. Smart patrol and swarm drones based on IoT are able to transmit information to authorities through WAN.

In power distribution, the power is stored in the distribution station, where various transformers are installed. Transformers play a crucial role in storing power and distributing it to residences, industries, etc. Here, monitoring the health status of the transformer is necessary for supplying continuous power, monitoring the oil level in the transformer, the health of the transformer, and the LT pillar for the RYB phase detector. Moreover, monitoring power theft and equipment management are the applications that are related to equipment. With the integration of IoT, the health monitoring of the transformer and other activities are remotely monitored through the NAN network. In the field of power consumption, the main application comes from homes because of the amount of power consumed by homes through refrigerators, washing machines, lights, and other possible appliances. An IoT-based smart grid in this field provides an opportunity for realizing the concept of smart home automation, where the appliances in the homes are automated. Smart home automation is the integration of smart sensors and actuator nodes that sense the environmental parameters and adjust lighting intensity and other appliances.

The data from the home regarding environmental parameters such as temperature, humidity, and air quality are transmitted to the home control unit through HAN. Another application of information management of EVs is the installation of renewable charging infrastructure for minimizing CO_2_ emissions. Generally, the charging system of an EV comprises three key components, namely the power supply system, monitoring system, and charging equipment.

AC chargers and DC chargers are the possible charging sources that are present in the charging equipment for charging appliances in the home (AC chargers) and vehicles (DC chargers). Here, IoT assists in managing and establishing an interface for managing the information related to billing of charging. The integration of GPS with EVs further assists the driver to locate the nearest and most appropriate charging station. Another application in power consumption is related to recording the utilization of power by homes and industries. Power utilization information is gathered in frequent time intervals; however, this mechanism has many shortages such as inaccurate information related to the amount of power consumed at a particular time. Advance meter infrastructure (AMI) is based on IoT and utilizes the wireless sensor network (WSN), optical programmable logic controller (OPLC), and PLC. AMI in the smart grid is able to gather real-time energy consumption with maximum reliability and the data can be utilized for performing the analysis of power consumption.

### 4.5. Microgrids

A small-size microgrid is a coalition of loads located in a particular distribution network feeder and is able to meet all demands by employing micro-scale generation resources such as photovoltaic panels, small-scale wind turbines, gas turbines, and micro-turbines [133]. Furthermore, in cases of lower demand, an energy storage facility may be utilized to supply the surplus production of micro-scale renewable energy sources. The most predominant kind of storage employed for battery units preserves the frequency stability of the microgrid. The microgrid design may have a remote structure, in particular, for remote areas or connected structures [134]. The connected mode is also recognized as the off-grid mode. The microgrid can trade the additional generation of internal resources to the grid in collective operation with an upstream network. In previous studies, hybrid systems deliver the cooperative functioning of micro-sources and storage facilities are proposed. Furthermore, some researchers have introduced the paradigm of interconnected microgrid dependency on the main grid. However, three problems are presented in microgrids, namely power quality, microgrid efficiency, and security of interconnected microgrids.

The inclusion of IoT will contribute to solving these issues, leading to the increased prevalence of the microgrid system that power system operators prefer [135]. Energy management must take place independent of the main grid in a microgrid. The upstream grid has no micro-source control and observation. The microgrid operator must make forecasts of uncertain micro-sources. The imbalances produced must be remedied by the internal storage device. In this context, however, the operator must enforce an unplanned load shedding or an unwanted cut-off due to the fact that such schemes have limited resilience capacities. The integration of IoT enhances the degree of observation and control of microgrid components by the main grid operator by taking into account the characteristics of all micro-sources, for the entire system generation, for connected microgrid operators [136]. IoT enables the establishment of better power system performance and more renewable energy resource penetration.

Furthermore, the microgrid operator will be able to make the microgrid more efficient and utilize renewable storage collaboration that corresponds to the actual energy market prices. Furthermore, real-time monitoring helps to improve the power quality of control systems [137]. Moreover, the mutual stability can affect two or more microgrids because of the incompatibility in scale. Any severe inequality in one can, therefore, risk other people’s security. To meet the demand continuously, frequency and voltage stability control systems are to be implemented [138]. This includes the utilization of the IoT infrastructure for internet-based environment; data from all sensors must be collected to inform the control devices in real time of the critical parameters [139]. In brief, data that correspond to all internal components of the microgrid can be shared with the upstream network operator via the implementation of the IoT infrastructure, which is currently not done [140]. This allows the main grid operator to monitor and control micro-sources and components of the microgrid. Moreover, the integration of IoT for microgrid operators encourages establishing a stable interconnection between the generation and storage unit for generating revenue. Additionally, IoT-based technologies facilitate an authority’s ability to sustain security in the interconnection of microgrids. Intelligent controllers enhanced by deep reinforcement learning (DRL) approaches are employed for the bottom-layer operation of each microgrid independently with bottom-up EI architecture and data-driven dynamical control strategy. The simulation has demonstrated that the suggested method reduces overall generation costs under the bottom-up design by 7.1% and 37%, respectively, when compared to conventional methods such as proportional integral and optimal power flow [141].

### 4.6. IoT in Services of Electrical Equipment

According to the UNSDG agenda, energy efficiency is one of the key elements for achieving sustainable development. Furthermore, energy efficiency provides long-term fiscal benefits by lowering the energy generation, emissions, and price of fuel imports and exports of the energy sector. An efficient study of real-time data in the energy supply chain is critical for improving energy efficiency and optimizing energy management. In energy, supply chain is categorized into three main components based on resource extraction to end-users, namely energy supply, energy transformation processes, and energy demand, which are available for energy use in industry, buildings, and transportation.

**Figure 10:**
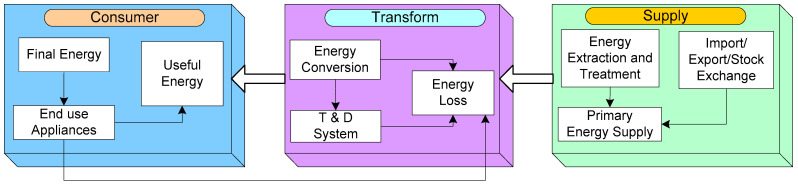
Energy Supply Chain.

Figure 10 depicts the three sections along with their respective elements and demonstrates the potential contribution of IoT to energy efficiency, energy demand reduction, and increasing the share of RESs. The energy consumption in smart cities is categorized into distinct parts, namely residential and commercial (offices, schools, and transport).

**Figure 11:**
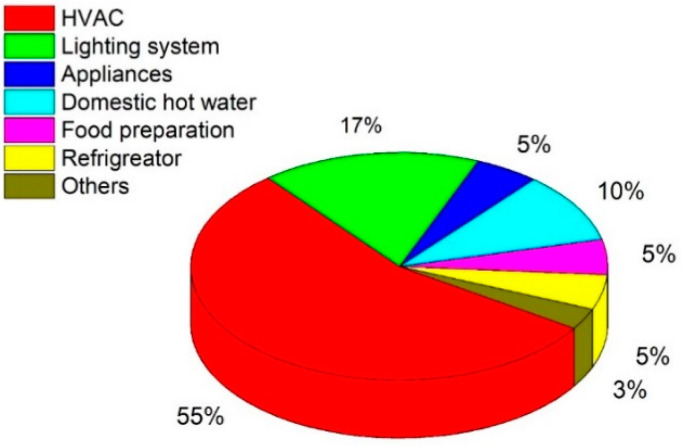
Proportion of residential energy consumption [142].

In the residential sector, energy consumption is possible due to lighting, cooking, refrigerators, heating, ventilation, and air conditioning (HVAC) and is clearly illustrated in Figure 11. The energy consumption is highest for HVAC systems in buildings. HVAC system management is critical for lowering the consumption of electricity. The progression in technology contributes significantly to controlling energy losses in HVAC systems [142]. When a vacant zone is discovered, certain steps can be taken to reduce energy consumption. HVAC systems, for example, can minimize operation in the vacant zone, resulting in substantial reductions in energy consumption and losses [142].

## 5. Blockchain for Energy Trading

**Figure 12:**
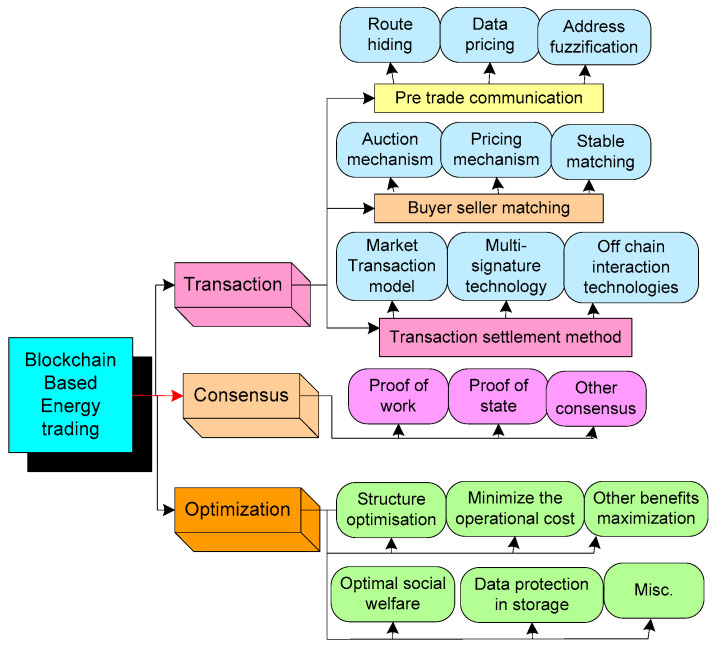
The architecture of blockchain-based trading.

Currently, the implementation of blockchain-based peer-to-peer energy trading has been a trend. Figure 12 illustrates the three-layer architecture of peer-to-peer (P2P) energy trading based on blockchain technology. Structured and unstructured P2P networks are the two broad organizational modes of P2P networks [143]. The unstructured peer location is chosen at random, eliminating the need for a centralized node search, but the query may not yield a result. We classify the initial subsection of energy transactions into three sub-sections: energy transactions, consensus, and optimization. Transactions are classified into three sub-sections, namely buyer–seller matching, pre-transaction communication, and transaction settlement [144].

**Figure 13:**
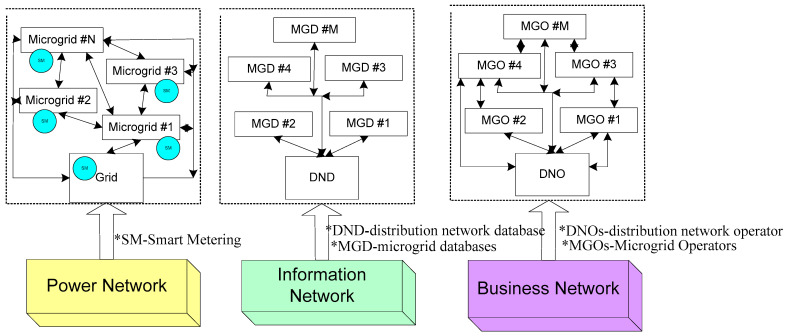
Structure of distribution network for peer-to-peer (P2P) energy trading.

The power network, business network, and information network are the part of super-network, which is shown in Figure 13. The power network is established with numerous networked microgrids in various geographical locations including distribution generation, electric vehicles, and general loads connected to distributed connection points. Each microgrid’s internal topology is also unique. Applications, communication devices, protocols, and information flow comprise the information network. 

The actual energy flow is logged in the distribution network database and microgrid databases through advanced energy meters that are installed in each microgrid. This layer also defines various control strategies for maintaining the quality and reliability of the power supply and regulating the power flow [145]. The business network is a peer-to-peer network that has been strengthened by a blockchain for the development of numerous business models that control energy trading. Microgrid operators are permitted to engage in peer-to-peer energy trading in the regional distribution network by following the energy market regulations [146].

Table 3 illustrates the recent studies that have implemented blockchain for energy trading. In the studies, it has been identified that the blockchain is implemented to provide extensive secured trading, auctioning, and demand management with transparency, immutability, and decentralization. Furthermore, the record of every single transaction at each node is stored in ledger format with a timestamp. The hash algorithm in the blockchain boosts security in the distribution of data. The consensus algorithms of a blockchain built on P2P networks deliver the secure distributed ledger. Once an update is included in the blockchain as a valid block, it is difficult to remove and manipulate it. In the majority of studies, the private blockchain is widely adopted for the implementation of P2P networks in energy trading.

## 6. Artificial Intelligence in Energy

**Figure 14:**
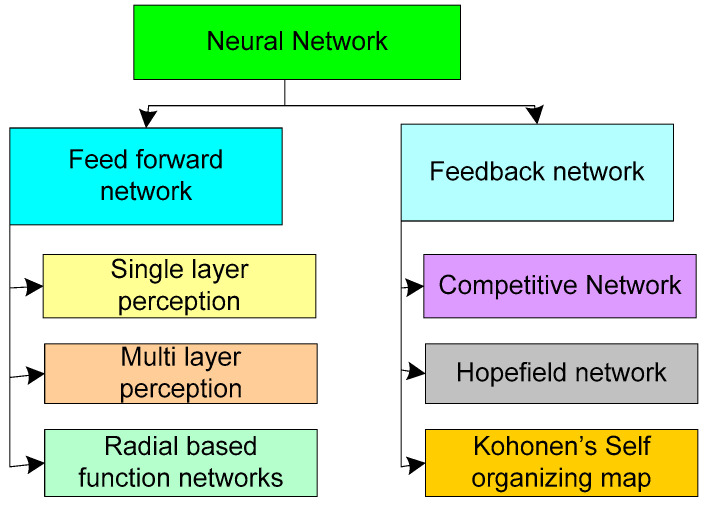
ANN Architecture.

AI is related to the integration of the intelligence of machines to perform tasks the same as humans for solving complex tasks. The advancement of AI has lowered the burden of manual computing. Furthermore, AI has outperformed in the region of complex tasks that are impossible for the average human’s intelligence. AI is used in a variety of fields, including database management, accounting, information retrieval, product design, production planning, distribution, economics and business, medicine, food quality monitoring, biometric and forensic analysis, and so on. AI is founded on various learning theories such as statistical learning, neural learning, evolutionary learning, and so on. Among these, neural learning is the most widely used in a variety of applications. The most basic neural learning method is ANN, represented in Figure 14.

Table 4 presents the previous studies that have implemented AI in energy. From the table, it has been identified that AI models such as the support vector regression technique are implemented to forecast the demand for the next day; the MLSTM technique is implemented to predict the smart grid network stability; and ANN controllers are implemented for the microgrid power balance. The AI-based Icosf control algorithm is implemented for increasing the power quality in microgrids and AIEM is utilized for the estimation of energy efficiency.

## 7. Discussion and Recommendations

Energy is an important source in the present generation for sustaining life on this planet. With the advancement of technologies, energy sources are carefully monitored and processed because of the limitation of resources and the wide increase in the population. In this study, we have discussed the significance of the Industry 4.0 revolution in the energy sector. We came up with limitations and recommendations in this study to enhance the distinct areas in the energy sector to accomplish the goal of Energy System 4.0 and the limitations and recommendations are as follows:

a. Dynamic communication

In IoT, wireless communication protocols play a crucial role in maintaining reliable transmission and connectivity with IoT-aided smart devices. An IoT-aided smart grid operates under critical environments, including some very severe conditions, such as the monitoring of power transmission lines [157]. Generally, in IoT, the communication protocols are selected based on several parameters, namely low power consumption, data rate, and long-range transmission [102]. In a smart grid, the information and activities are considered critical because of the delay in the transmission of information related to power management, and appliance scheduling leads to failure. Thus, the utilization of wireless communication protocol plays a crucial role, as the information needs to be transmitted to a long range within a short interval of time. Therefore, it is important to consider the requirements for establishing hybrid communication technologies and signaling coverage under adverse environmental conditions [158].

b. Energy sources for end nodes

Fundamentally, the end nodes are the backbone for sensing the distinct parameters of smart grid sub-systems (transmission, distribution, and utilization) using sensors and camera nodes. However, the power consumption of these end nodes plays a crucial role, as the end nodes are working on batteries [159]. To overcome this challenge, the end nodes need to be inbuilt with an energy storage unit and, additionally, an energy harvesting-supported design needs to be implemented in end nodes [160]. Here, extensive research is required on building long-term-capacity batteries for the end nodes.

c. Machine learning (ML) in end nodes of smart grid

The integration of ML in end nodes of the smart grid enables forecasting of demand, power utilization, and power prices. Fundamentally, ML is able to learn from previous events and enhance decision making. In the smart grid, the prediction of electrical load is crucial as it depends on time and weather data [161]. The precise estimation of electrical load will assist the electrical authorities in managing electricity production, management, control, and operations [162]. The prediction of electrical load for future load values is possible by analyzing the obtained data from smart meters [163].

d. Blockchain in smart grid

Blockchain is a distributed ledger technology that performs distributed transactions by eliminating central entities [164]. In energy systems, many rapid changes are undergoing with the utilization of renewable energy sources. In renewable energy sources, the prediction of events is challenging because it depends upon the weather conditions. To overcome this challenge, flexible ways are to be implemented to assure safe operation and management [165]. Such methods comprise demand response, fast-acting supply, and energy storage devices. To implement these flexible ways, a significant investment is required, and this investment needs to perform on a secure decentralized platform. Here, the blockchain will assure secure and decentralized transactions between energy utilities and consumers [166].

e. Green IoT

The energy consumption of IoT devices is a substantial challenge in the IoT ecosystem, particularly in the context of extensive distribution in the near future [167]. A large amount of energy is required to power a billion devices that are connected to the internet. The huge amount of IoT devices generates a large amount of electronic waste [168]. Low-carbon and energy-efficient communication are possibilities for minimizing the environmental impact. Green IoT (G-IoT) can achieve the objective of low carbon emissions [169]. G-IoT offers energy-efficient features throughout its life cycle, including production, design, deployment, and finally, disposal [170]. The G-IoT cycle is utilized in a variety of IoT applications. In radio frequency identification (RFID) tags, the size of RFID tags is reduced in order to reduce the amount of material in each of the RFID tags, which are difficult to recycle [171].

f. Virtual Power Plant with Metaverse

Virtual power plants (VPPs) are sustainable alternatives for tackling the smart energy grid’s decarbonization and energy efficiency goals. Metaverse strives to optimally combine and coordinate energy production with storage and consumption resources with controllable loads to satisfy renewable inclusion and energy cost minimization requirements [172]. VPPs essentially collect resources to connect energy markets, plan their functions to aggregate energy profiles, give energy services, ensure a stable supply, and provide demand response services. A decentralized VPP optimization system built on the highest blockchain infrastructure can be used to provide a verifiable, transparent, and trustworthy management structure for the VPP’s dependable energy service delivery.

g. Big data for energy analytics

Energy analytics has advanced rapidly in the last decade due to advancements in big data and machine learning. Energy data have grown enormously in several areas and domains as a result of this advancement in energy analytics [173]. Energy computing approaches have been utilized to handle data processing in a variety of disciplines, particularly in energy data. It also allows for power outage prediction, detection, and prevention, as well as smart load management and preventive asset management.

h. Digital twins in smart grid modeling

Advanced manufacturing systems are incorporating digital twins to monitor and optimize industrial operations. Energy consumption and distribution are highly unpredictable, and there is an evolving requirement for forecasting energy demands as well as distributing energy in the most efficient manner feasible such that manufacturing processes are not hindered [174]. Future work focuses on the interconnection of the functional blocks that comprise the smart grid to expand and computerize the process of managing power sources and customer blocks.

i. Standardization of IoT for smart grid

IoT employs distinct kinds of technologies with different standards for establishing an interconnection between a single device to many numbers of devices. However, the instability among IoT devices creates a challenging task because IoT utilizes different standards [175,176]. Every network protocol and wireless communication protocol comprises distinct standards for data transmission [177]. Moreover, the security and privacy of the data are also significant elements that need to be considered [178]. To address the challenge in the standardization of IoT-based energy systems, the strategy is to recognize a system of systems with cooperation so that all actors can access and utilize these systems equally. Another strategy is for collaborating entities to construct open-information models and standards-based protocols. As a result, standards will be freely and publicly accessible [179,180].

## 8. Conclusions

Emissions from energy need to decline by 55% by 2030; however, emissions are set to remain around the same level for the next three years. The transformation in the energy industry leads toward managing energy sources sustainably and reliably. The evolution of digitalized technology including IoT transformed the traditional energy grid into the smart grid.

In this article, we have discussed the significance of Industry 4.0 enabling technologies (IoT, AI, edge computing, blockchain, big data, AR/VR, digital twins, and metaverse) in the field of the energy system for enhancing the activities in the four sub-systems: smart grid, microgrid, electrical equipment, and energy trading. Initially, the article discussed the different enabling technologies of Industry 4.0 for bringing the energy system onto the digital network to strengthen its monitoring and enhance energy efficiency. Prior to the discussion of these technologies’ integration into energy systems, the study addressed the different obstacles that exist in the energy sector and also addressed the need for IoT integration. This is because IoT is the core technology of Industry 4.0 to connect physical things to the digital network.

Following this, the article discussed the integration of IoT for energy generation transmission, distribution, and consumption. In which, the article addressed the energy generation from renewable energy sources (wind, solar, and thermal) that are prioritized for implementation to minimize carbon emissions. Resource generation management: realizing the output status of the generators, transformers, tap changers, and electrical status of transmission lines including perturbations; and improper monitoring of energy consumption in the domestic and other areas are the problems identified in the four sub-systems of energy. We discussed the studies with an IoT-based architecture perspective for overcoming the problem and implementing smart and real-time monitoring. Along with this, fog- and edge-assisted cloud architecture for the smart grid are also presented with applications.

This study also analyzed and identified the application of AI and blockchain in the energy system, in which AI is majorly implemented in demand side management, forecasting generation for the next day, and also in forecasting smart grid network stability. Blockchain technology is distributed ledger technology that empowers the creation of a smart contract with hash cryptography. This technology inspires the implementation of energy trading in a transparent, secure, and immutable manner. It has been identified that the blockchain is majorly utilized in energy trading for households, industries, etc.

Based on the analysis in the article, the study has formulated vital recommendations that can be applied in future work. These recommendations are formulated with the importance of technical capability in resolving the problems in the energy system with efficiency and sustainability. It is challenging to implement all these technologies in the energy system. However, to enhance the infrastructure of the energy system, this study suggested recommendations with individual technologies. The implementation of digital twins and Metaverse are some of the latest technologies that have already gained significant attention for smart grid modeling and the development of virtual power plants.

Regarding the highlights of this study, we discussed the integration of IoT, blockchain, and AI with an architectural perspective in the different sub-systems of the energy system for achieving real-time monitoring with intelligence and predictive analytics. Based upon analysis, the article presented vital recommendations for individual technology integration in energy for multiple applications. A limitation of the current study is that it has addressed only three enabling technologies of Industry 4.0. The study will be further extended with the discussion of other enabling technologies as a part of future work.

## Figures and Tables

**Table 1:** Applications of IoT for the energy sector.

Application	Field	Explanation	Advantages
Energy democratization	Regulation	Establishing peer-to-peer electricity trading for small end-users	Creates awareness of energy usage and efficiency with a centralized supply chain.
Preventive maintenance	Utility companies and upstream oil and gas industry	Utilizing sensors/ cameras for detecting faults and leakage	Minimizing the risk of failure, maintenance downtime, and enhancing safety.
Energy storage and analytics	Utility companies and industrial distributors	Evaluating the market and possibilities for implementing energy storage in the system	Minimizing the imbalance between demand and supply, maximizing the flexibility in energy storage and profitability.
Digitalized power generation	System operators and utility companies	Controlling many generation units	Enhancing the assets utilization and management and minimizing the risk of blackout.
Smart grid	Grid management	Establishing and providing intelligence to the grid using ICT and big data	Enhancing the energy efficiency and security of supply, minimizing the necessity of the backup supply capacity.
Network management	Management and operation of the grid	Employing big data at the distinct point for managing the grid more optimally	Checking the weak points in the grid for avoiding blackouts
Microgrids	Electricity grid	Managing the independent grid from the central grid	Establishing interoperability between microgrid and central grid. Charging stable price to consumers connecting to microgrid
Integrated control of the electric vehicle (EV) fleet	Management and operation of the grid	Monitoring the charging data of the stations and analyzing the charge and discharger state of EVs.	Evaluating the impact of EV on load. Recognizing the places for the establishment of new charging stations
Demand response	Commercial/ industry/residential	Shielding, shifting, and leveling	Minimizing the demand at peak times leads to a reduction in grid congestion
Advanced metering infrastructure (AMI)	End clients	Sensors and devices are installed at the consumer site for obtaining data on load and temperature	Facilitates evaluation of load variations at a distinct time interval and detects the areas for enhancing energy efficiency
Battery energy management	End clients	Performing data analytics for triggering the battery at the appropriate time	Enhancing the energy efficiency by implementing the optimal strategy of charging and discharging the battery
Home automation	End clients	remote monitoring and controlling of devices and home appliances	Time saving and energy, improving knowledge on energy use, enhanced integration of disturbed generation and storage systems

**Table 2:** Technical specifications of distinct wireless communication technologies [54].

Parameter/Technology	Transmission Distance	Bit rate	Lifetime of Battery	Security	Infrastructure Cost	Implemented for
Satellite	>1500 km	100 kbps	High	High	Costly	Wind and solar plants
Zigbee	≤100 m	250 kbps	5–10 years	Low	Low	Energy meter for renewable energy
Bluetooth	≤50 m	1 Mbps	Low	High	Low	Home automation
Weightless	<5 km	100 kbps	Low	High	Low	Energy meter
Sigfox	≤50 km	100 bps	7–8 years	High	Moderate	Electric plugs
LTE-M	≤200 km	0.2–1 Mbps	7–8 years	High	Moderate	Energy meter
NB-IoT	≤50 km	≤100 kbps	1–2 years	High	Low	Grid communication
LoRA	≤50 km	0.3–38.4 kbps	8–10 years	High	Low	Lighting

**Table 3:** Recent studies of Blockchain for energy trading.

Research	Objective	Blockchain Type
[147]	P2P trading in retail electricity markets using a blockchain-based architecture	Ethereum private chain
[148]	P2P scheme centered on smart contracts for double energy auctions and reserve trading	Ethereum
[149]	Transparent and Decentralized P2P energy trading to lower the grid’s energy generation and administration load simultaneously improving profit for both consumers and prosumers.	Ethereum blockchain
[150]	Decentralized ledge integration in the electricity marketplace for the smart grid context to enable stakeholder trust and traceability	Ethereum blockchain
[151]	Power trading system that is decentralized and uses distributed ledger technology.	Corda-based private permissioned distributed ledger.

**Table 4:** AI in the Energy.

Research	Objective	AI
[152]	Demand-side management forecasts dispersed generation for the next day.	Support Vector Regression technique
[153]	Forecast the smart grid network’s stability.	Multidirectional Long Short-Term Memory (MLSTM) technique
[154]	Preserve the microgrid’s power balance.	ANN controllers
[155]	Power sharing and increased power quality in intelligent microgrid systems	AI-based Icosf control algorithm
[156]	Estimating the effects of energy efficiency and renewable energy on the economy	AI-based useful evaluation model (AIEM)

## Data Availability

Not applicable.
